# Evidence that xylazine disrupts skin homeostasis by acting on epithelial cells through the kappa opioid receptor

**DOI:** 10.1242/dmm.052600

**Published:** 2026-03-31

**Authors:** Tanner F. Robertson, Adam Horn, Frances M. Smith, Anna Huttenlocher

**Affiliations:** ^1^ Department of Medical Microbiology and Immunology, University of Wisconsin School of Medicine and Public Health, Madison, WI 53706, USA; ^2^ Department of Pediatrics, University of Wisconsin-Madison School of Medicine and Public Health, Madison, WI 53792, USA

**Keywords:** Xylazine, Wounds, Epidermis, Keratinocytes, Opioid

## Abstract

The veterinary sedative and alpha-2 adrenergic receptor (α2AR) agonist xylazine, found in the illicit opioid supply, is associated with cutaneous wounds in humans. Here, we developed a larval zebrafish model of xylazine-induced tissue damage to investigate the mechanisms by which xylazine affects the skin. Xylazine treatment caused keratinocyte extrusion, tissue-wide skin contraction and disruption of basal keratinocyte cell–cell interactions in zebrafish larvae. Notably, other α2AR agonists did not recapitulate most of these effects. Xylazine was recently described as a kappa opioid receptor (κOR) agonist, and we found that both xylazine and a separate κOR agonist acted directly on epithelial cells to drive cellular contraction and disrupt tissue homeostasis. Our model suggests that xylazine disrupts skin homeostasis through a direct mechanism involving epithelial cells and κOR, which may be of importance for the treatment of these wounds.

## Teaser

**Summary:** Xylazine, a common adulterant in illicit opioids, causes severe skin wounds. Using a zebrafish model, we show that xylazine acts directly on keratinocytes through the kappa opioid receptor to disrupt epithelial homeostasis.

## INTRODUCTION

Xylazine is an alpha-2 adrenergic receptor (α2AR; also known as ADRA2) agonist and veterinary sedative found in the illicit opioid supply in the United States. Xylazine is colloquially known as ‘tranq’, and its use is associated with skin damage, ranging from superficial irritation to severe necrosis that exposes the underlying soft tissue and bone (Bishnoi et al., 2023; D'Orazio et al., 2023; Ehrman-Dupre et al., 2022; McFadden et al., 2024; Rose et al., 2023; Rubin, 2023; Smith et al., 2023; Tosti et al., 2024). Between 2015 and 2022, the prevalence of xylazine in fentanyl samples and xylazine-induced wounds increased across the United States, prompting the Office of National Drug Control Policy to declare xylazine an emerging threat in April 2023. In patients with confirmed xylazine exposure, wounds most commonly occur in the extremities, particularly at extensor surfaces (Lutz et al., 2025), although distal wounds have also been reported (Zagorski et al., 2023). Some individuals have noted that wounds often appear when they inadvertently miss the vein during an injection, which likely results in prolonged exposure to high concentrations of xylazine in the surrounding tissue (Malayala et al., 2022; McNinch et al., 2021).

The mechanism by which xylazine causes these wounds remains unknown, although several hypotheses have emerged. The most prominent is that xylazine, by acting on smooth muscle cells through α2AR, causes peripheral vasoconstriction, which results in poor perfusion and oxygenation of the skin that drives necrosis and tissue damage (McNinch et al., 2021). Other studies have examined whether the low pH of xylazine-containing drug samples may contribute wound healing defects, perhaps by affecting blood vessel integrity (Ciccarone et al., 2025). However, our understanding of how xylazine affects the skin is limited by the lack of any published animal models that can recapitulate these effects in a laboratory setting.

To bridge this gap, we developed a larval zebrafish model of xylazine-induced skin damage. We found that xylazine exposure through bath immersion replicates the major effects seen in humans including sedation, bradycardia and skin damage. We took advantage of the ability to perform live imaging of keratinocyte dynamics in zebrafish larvae to dissect how xylazine alters skin homeostasis. We found that xylazine has three major effects on the skin: superficial keratinocyte extrusion, tissue-wide skin contraction and disruption of basal keratinocyte cell–cell interactions. Xylazine is a well-known α2AR agonist, but recent data indicate that it activates several other receptors, including the kappa opioid receptor (κOR; also known as OPRK1) (Bedard et al., 2024; Floresta et al., 2025). Here, we provide evidence that xylazine-induced keratinocyte extrusion is mediated by α2AR, whereas skin contraction and basal keratinocyte disruption is mediated by κOR. Our findings support a model in which xylazine, by acting as a dual receptor agonist, can exert pleiotropic effects on the skin. Finally, we provide evidence that xylazine can act directly on epithelial cells through κOR to damage the skin. Although our results are not mutually exclusive with the prevailing vasoconstriction hypothesis of xylazine-induced wounds, they point to an alternative mechanism by which xylazine may be causing skin damage.

## RESULTS

### Xylazine causes sedation, bradycardia and skin deterioration in zebrafish larvae

**Fig. 1.**
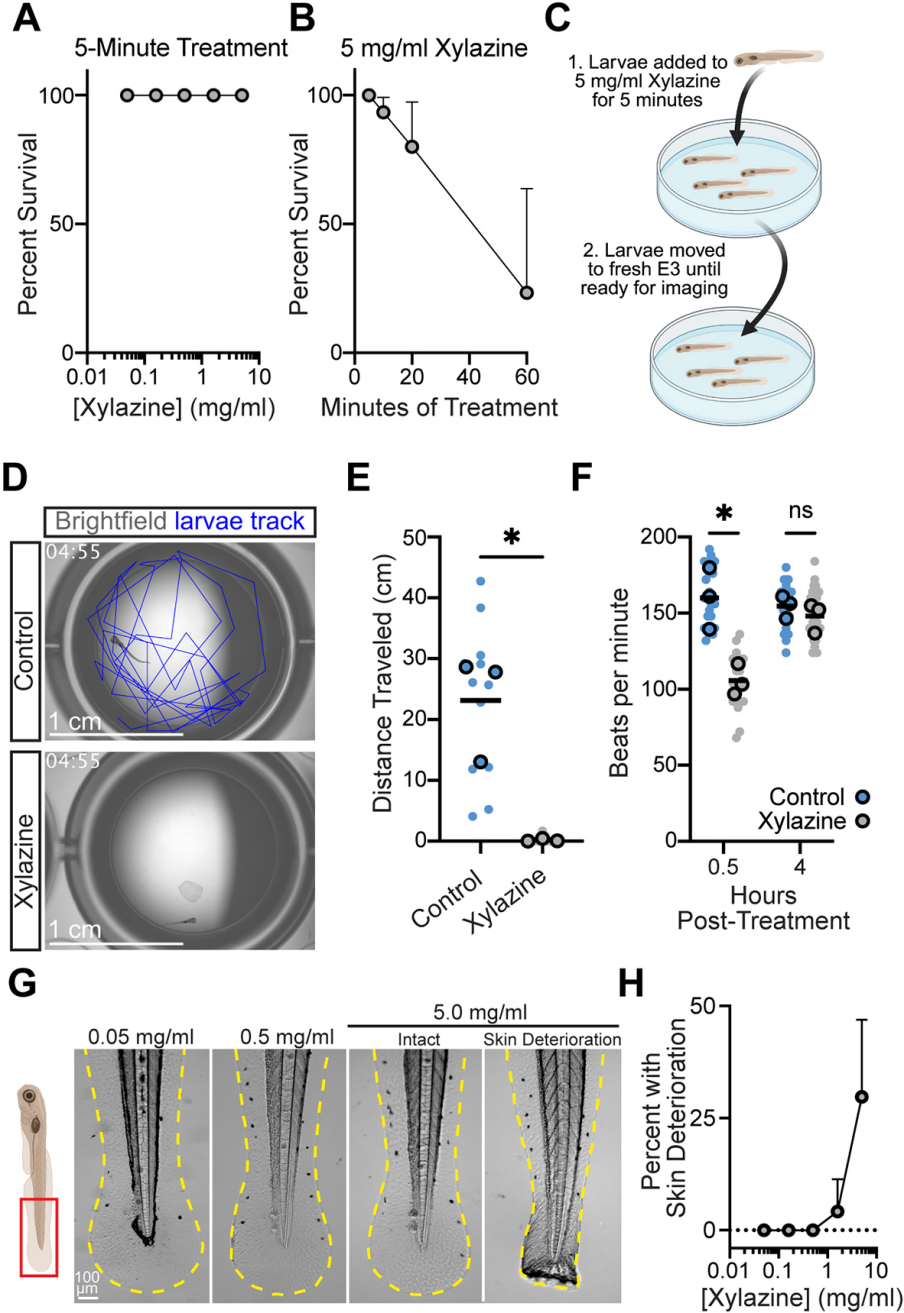
**Xylazine treatment causes sedation, bradycardia, and skin deterioration in larval zebrafish.** (A) The percentage survival of larval zebrafish treated with increasing concentrations of xylazine for 5 min across three independent experiments (*n*=24 total larvae per condition). (B) The percentage survival of larval zebrafish treated with 5 mg/ml xylazine for the indicated amount of time across three independent experiments with eight to ten larvae per condition per experiment (total larvae for each timepoint: 5 min, 28; 10 min, 30; 20 min, 29; 60 min, 29). (C) A schematic of the xylazine treatment approach used throughout this figure. Larval zebrafish were treated with 5 mg/ml xylazine for 5 min and then moved to fresh E3 medium until they were ready for imaging. (D) Representative brightfield images with the overlaid larval swim tracks (blue) of control and xylazine-treated fish in a 24-well plate, showing the sedation of xylazine-treated fish. Time in mm:ss. Scale bars: 1 cm. (E) The total distance traveled by control and xylazine-treated larvae over the course of a 5-min movie. The averages of three independent experiments (large dots) representing 12 individual larvae (four larvae per experiment, small dots) were compared using a two-tailed nested *t*-test. *P=0.0106. (F) Heart rate in beats/min of control and xylazine-treated fish at 0.5 and 4 h post-treatment, showing that xylazine treatment causes transient bradycardia. The averages of three independent experiments (large dots) representing 24 individual larvae (eight larvae per experiment, small dots) were compared using two-tailed nested *t*-tests. ns, not significant; *P=0.0143. (G) Representative images of the post-anal fin fold region of zebrafish larvae treated with increasing concentrations of xylazine, showing the skin deterioration resulting in the loss of overall fin shape seen in some larvae. (H) Quantification of the percentage of larvae with skin deterioration following treatment with increasing doses of xylazine from three independent experiments with eight to 12 larvae per condition per experiment (total larvae per dose: 0.05 mg/ml, 27; 0.16 mg/ml, 29; 0.5, 1.6 and 5.0 mg/ml, 30). Data are presented as mean±s.d. where error bars are shown (A,B,H). Large dots in SuperPlots (E,F) represent experiment means.

We began by screening for a xylazine dose and treatment duration that resulted in sedation and bradycardia in larval zebrafish at 5 days post-fertilization (dpf) without causing death. We found that all larvae survived a 5-min bath exposure of xylazine at a dose up to 5 mg/ml (Fig. 1A). An increasing number of larvae died as the treatment duration was increased to 10, 20 and 60 min (Fig. 1B), so we limited our initial experiments to a 5-min treatment with 5 mg/ml xylazine (Fig. 1C). To determine whether xylazine causes sedation, we monitored control and xylazine-treated larvae in a 24-well plate swim assay in the 1-h period immediately after treatment. We found that xylazine-treated fish showed clear sedation, as judged by the lack of swimming over the imaging period (Fig. 1D,E; Movie 1). We next manually counted heartbeats/min at 0.5 and 4 h post-xylazine treatment and found that xylazine caused transient bradycardia that was recovered by 4 h post-treatment (Fig. 1F). Most importantly, we found that this xylazine treatment approach resulted in severe skin deterioration characterized by a loss of overall fin shape in ∼30% of larvae (Fig. 1G,H). Collectively, these data indicate that zebrafish larvae can recapitulate the major effects of xylazine seen in humans: sedation, bradycardia and skin damage.

### Xylazine treatment triggers extrusion, tissue-wide contraction and disruption of basal keratinocyte adhesions

**Fig. 2.**
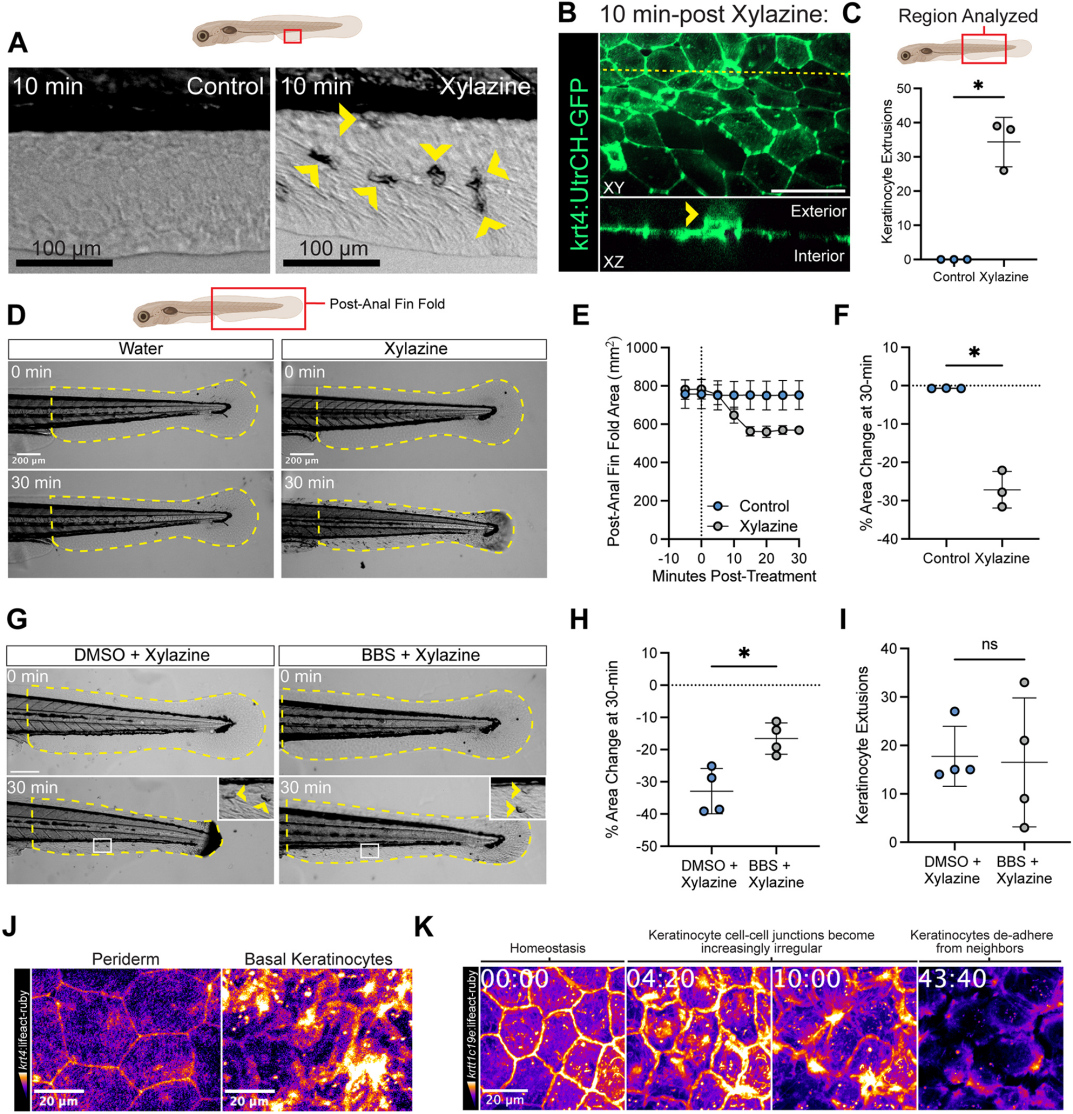
**Xylazine treatment triggers extrusion, tissue-wide contraction and the disruption of basal keratinocyte adhesions.** (A) Timelapse brightfield images of the skin of larval zebrafish, showing apparent keratinocyte extrusions (yellow arrowheads) at 10 min post-treatment with xylazine but not in control larvae. Scale bars: 100 µm. (B) Orthogonal projection of the skin of a Tg(*krt4*:UtrCH-GFP) keratinocyte reporter larva at 10 min post-treatment with xylazine, showing keratinocyte extrusion from the epidermis (yellow arrowhead). The yellow line in the *xy* image denotes the plane of orthogonal projection. Scale bar: 50 µm. Representative of ten larvae from three independent experiments. (C) The number of cumulative keratinocyte extrusions that develop over the post-anal fin fold of control and xylazine-treated larvae between 0 and 30 min, with the averages from three independent experiments (*n*=3 larvae total; one larva per experiment) compared with a two-tailed Welch's *t*-test. *P=0.0145. (D) Timelapse brightfield imaging of the post-anal fin fold (outlined in yellow dashed lines) of control and xylazine-treated (5 mg/ml) larvae, showing tissue-wide contraction. Representative of three independent experiments. Scale bars: 200 µm. (E,F) The change in 2D area from three independent experiments (*n*=3 larvae total; one larva per experiment) of the post-anal fin fold over time (E) or at 30 min post-treatment (F) with xylazine (5 mg/ml). The change in 2D area at 30 min post-treatment was compared in F with a two-tailed Welch's *t*-test. *P=0.0107. (G) Timelapse brightfield imaging of the post-anal fin fold (outlined in yellow dashed lines) of zebrafish larvae pre-treated with 100 µM *para*-aminoblebbistatin (BBS) or an equal volume of DMSO for 30 min and then subsequently treated with xylazine (5 mg/ml), with insets and yellow arrowheads highlighting keratinocyte extrusions. Time in mm:ss. Scale bar: 200 µm. (H,I) The change in 2D area from four independent experiments of the post-anal fin fold (H) and the number of cumulative keratinocyte extrusions (I) at 30 min post-treatment from four independent experiments (*n*=4 total larvae per condition; one larva per experiment), both compared with a two-tailed Welch's *t*-test. *P=0.0110 (H); ns, not significant (I). (J) Confocal imaging of the periderm and basal keratinocyte layers, identifiable in different *z*-layers, in Tg(*krt4*:LifeAct-mRuby) larvae at 30 min post-xylazine treatment, showing the selective disorder of the basal layer. Representative of three independent experiments. Scale bars: 20 µm. (K) Timelapse imaging of the basal keratinocyte F-actin dynamics in Tg(*krtt1c19e*:LifeAct-mRuby) larvae following xylazine treatment, showing the disruption of clear intercellular adhesions and the eventual de-adherence of neighboring cells. Representative of three independent experiments (*n*=9 total larvae). Time in mm:ss. Scale bar: 20 µm. Data are presented as mean±s.d. in all graphs.

To better characterize the effect of xylazine on the skin, we performed live imaging of zebrafish larvae following treatment with xylazine. We consistently observed three primary phenomena following xylazine treatment. First, xylazine treatment resulted in the rapid extrusion of keratinocytes from the epidermis visible by both brightfield microscopy (Fig. 2A,C; Movie 2) and in orthogonal projections of Tg(*krt4*:UtrCH-GFP) larvae (labeling keratinocyte F-actin) imaged by confocal fluorescence microscopy (Fig. 2B). Here, the keratinocyte could be seen extruding out of the epidermis and into the exterior space (Fig. 2B, yellow arrowhead in orthogonal *xz* projection). These keratinocyte extrusions were not observed when we treated fish with an equal volume of reverse-osmosis (RO) water (control), the solvent used for dissolving xylazine (Fig. 2A,C). Second, xylazine treatment caused the entire post-anal fin fold to contract, resulting in a sharp drop in 2D area between 5 and 15 min post-treatment that was maintained to at least 30 min (Fig. 2D-F; Movie 2). We did not observe a similar change in either the length or 2D area of the post-anal notochord and somite region in the same larvae (Fig. S1A,B), suggesting that xylazine was preferentially acting on the skin. We hypothesized that this tissue-wide contraction may be driven by actomyosin contractility, so we pre-treated larvae with the myosin inhibitor blebbistatin (BBS) or DMSO (vehicle) and subsequently treated with xylazine. Here, we found that BBS treatment significantly reduced this tissue-wide contraction without affecting the number of keratinocyte extrusions in the same larvae (Fig. 2G-I). These data indicate that the xylazine-induced contractions and extrusions are controlled by different cytoskeletal processes.

We next investigated how keratinocytes responded to xylazine treatment. The larval zebrafish epidermis is composed of two layers of keratinocytes – the basal layer and periderm. The F-actin of keratinocytes in both layers are visible in Tg(*krt4*:LifeAct-mRuby), and each layer can be distinguished by focusing on different *z*-heights. We found that, following 30 min of xylazine treatment, the periderm maintained grossly normal architecture with F-actin concentrated at the intercellular adhesions (Fig. 2J), consistent with published descriptions of this population under homeostasis (Lee et al., 2014). In contrast, the basal keratinocyte layer immediately subjacent appeared highly disorganized, with the cell boundaries hard to determine (Fig. 2J), compared to how basal keratinocytes appear under homeostasis (Fig. 2K, leftmost image). To investigate how xylazine was affecting the basal keratinocyte layer in real time, we performed live imaging of F-actin dynamics specifically in the basal layer by using the Tg(*krtt1c19e*:LifeAct-mRuby) reporter zebrafish line. Between 0 and 10 min post-treatment with xylazine, basal keratinocyte intercellular junctions became increasingly irregular, and, eventually, keratinocytes appeared to de-adhere from their neighboring cells (Fig. 2K; Movie 3). To determine the fate of these de-adhered keratinocytes, we simultaneously visualized basal keratinocyte GFP and keratinocyte F-actin with Tg(*krt4*:acGFP; *krtt1c19e*:LifeAct-mRuby) larvae post-xylazine treatment (Fig. S2A). We found that cytoplasmic projections eventually emerged from these keratinocytes to form new contacts with neighboring cells that were maintained to at least 2 h post-treatment (Fig. S2A). Notably, these new contacts appeared to have reduced F-actin compared to standard keratinocyte intercellular adhesions (Fig. S2A). In summary, xylazine treatment drives three major responses in the skin of larval zebrafish: keratinocyte extrusion, tissue-wide contraction and the disruption of basal keratinocyte cell–cell interactions.

### Xylazine-induced keratinocyte extrusion and skin contraction are mediated by α2AR and κOR, respectively

**Fig. 3.**
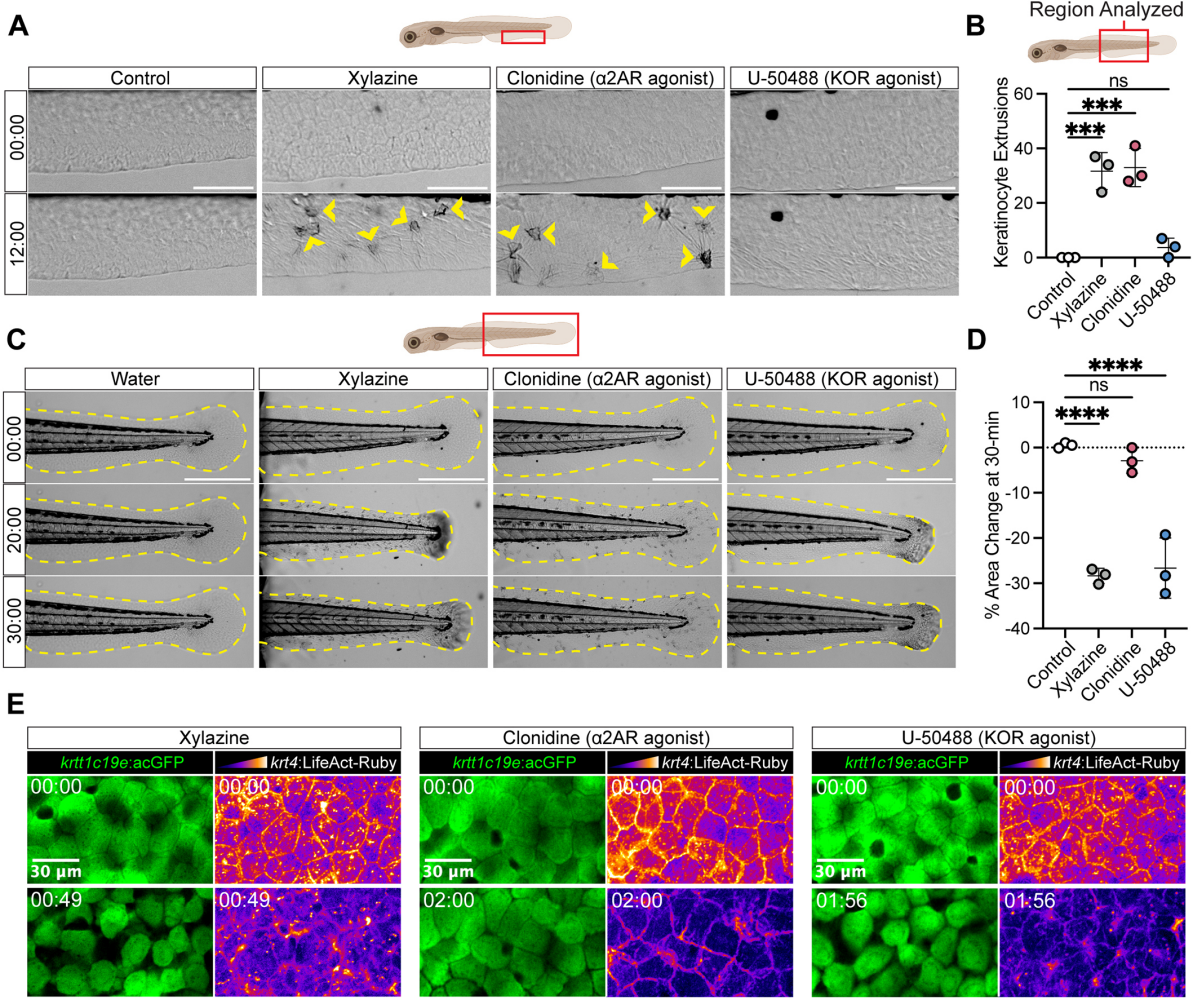
**Xylazine-induced keratinocyte extrusion and skin contraction can be replicated by alpha-2 adrenergic receptor (α2AR) and kappa opioid receptor (κOR) agonists, respectively.** (A,B) Timelapse brightfield imaging (A) and associated quantification (B) of keratinocyte extrusion (yellow arrowheads) in control larvae and those treated with xylazine (5 mg/ml), clonidine (α2AR agonist, 5 mg/ml) or U-50488 (κOR agonist, 1 mM). Time in mm:ss. Scale bars: 100 µm. The average keratinocyte extrusion of each condition from three independent experiments (*n*=3 larvae total per condition; one larva per condition per experiment) were compared to control using an ordinary one-way ANOVA with Dunnett’s correction for multiple comparisons. ns, not significant; ***P(control versus xylazine)=0.0002, ***P(control versus clonidine)=0.0001. (C,D) Timelapse brightfield imaging (C) and associated quantification (D) of the post-anal fin fold (yellow dashed outline) 2D area in control zebrafish and those treated with xylazine, clonidine or U-50488 as described in A,B. Time in mm:ss. Scale bars: 500 µm. The average change in 2D area in each condition at 30 min post-treatment from three independent experiments (*n*=3 larvae total per condition; one larva per condition per experiment) was compared to control using an ordinary one-way ANOVA with Dunnett’s correction for multiple comparisons. ns, not significant; ****P(control versus xylazine and control versus U-50488)<0.0001. (E) Timelapse imaging basal keratinocyte GFP and keratinocyte LifeAct in Tg(*krtt1c19e*:acGFP; *krt4*:LifeAct-mRuby) zebrafish larvae following treatment with xylazine (5 mg/ml), clonidine (5 mg/ml) or U-50488 (1 mM), showing the disruption of basal keratinocyte interactions following xylazine and U-50488, but not clonidine, treatment. Representative of three independent experiments (*n*=3 larvae total per condition; one larva per experiment). Time in mm:ss. Scale bar: 30 µm. Data are presented as mean±s.d. in all graphs.

Xylazine is used in veterinary medicine as an α2AR agonist, and a recent study found that it is also a full agonist of the kappa opioid receptor (κOR) (Bedard et al., 2024). We therefore investigated which receptor(s) is mediating the xylazine-induced skin disruption observed in our model. Clonidine, a separate α2AR agonist that is structurally similar to xylazine, resulted in keratinocyte extrusion to the same degree as xylazine treatment in larval zebrafish (Fig. 3A,B). In contrast, the κOR agonist U-50488 did not cause a significant increase in keratinocyte extrusion (Fig. 3A,B; Movie 4). These data indicate that xylazine likely drives keratinocyte extrusion by activating α2AR. We were surprised to find that, although clonidine could elicit robust keratinocyte extrusion, it did not drive any significant skin contraction in these experiments (Fig. 3C,D; Movie 4). However, U-50488 replicated the tissue-wide contraction induced by xylazine in zebrafish larvae (Fig. 3C,D; Movie 4). These data indicate that xylazine likely drives tissue-wide contraction by activating κOR.

To investigate whether either agonist could disrupt basal keratinocyte interactions as observed following xylazine treatment (Fig. 2G,H), we treated Tg(*krtt1c19e*:acGFP; *krt4*:LifeAct-mRuby) larvae, which express GFP specifically in basal keratinocytes and LifeAct-mRuby in all keratinocytes, with xylazine, clonidine and U-50488. We limited our imaging specifically to the basal keratinocyte layer to exclude LifeAct signal from the periderm, which did not undergo the same disruption (Fig. 2J). We found that U-50488, but not clonidine, resulted in the same disorganization of basal keratinocyte junctions observed following xylazine treatment, which was apparent by the detachment of keratinocytes visible in the GFP channel and the loss of clear intercellular junction visible in the LifeAct channel (Fig. 3E). Collectively, these data suggest that the dual agonist properties of xylazine enable it to exert distinct effects on the skin by simultaneously activating both α2AR and κOR.

### Xylazine and U-50488 act directly on epithelial cells to drive skin damage

Finally, we sought to determine which cell type(s) xylazine is affecting in the skin and through which receptor(s). The major hypothesis for why xylazine causes skin damage in humans is that it results in peripheral vasoconstriction, resulting in poor perfusion of the skin and, eventually, tissue damage (Kacinko et al., 2022; Liu et al., 2007; Oscherwitz et al., 2024; Rose et al., 2023). These vasoconstrictive effects of xylazine are thought to be mediated primarily by smooth muscle cells, which highly express α2AR (Chotani et al., 2004; Gambardella et al., 2023; Richman and Regan, 1998). In our model, xylazine disrupted keratinocyte homeostasis within minutes, leading us to hypothesize that this drug might act directly on epithelial cells themselves.

**Fig. 4.**
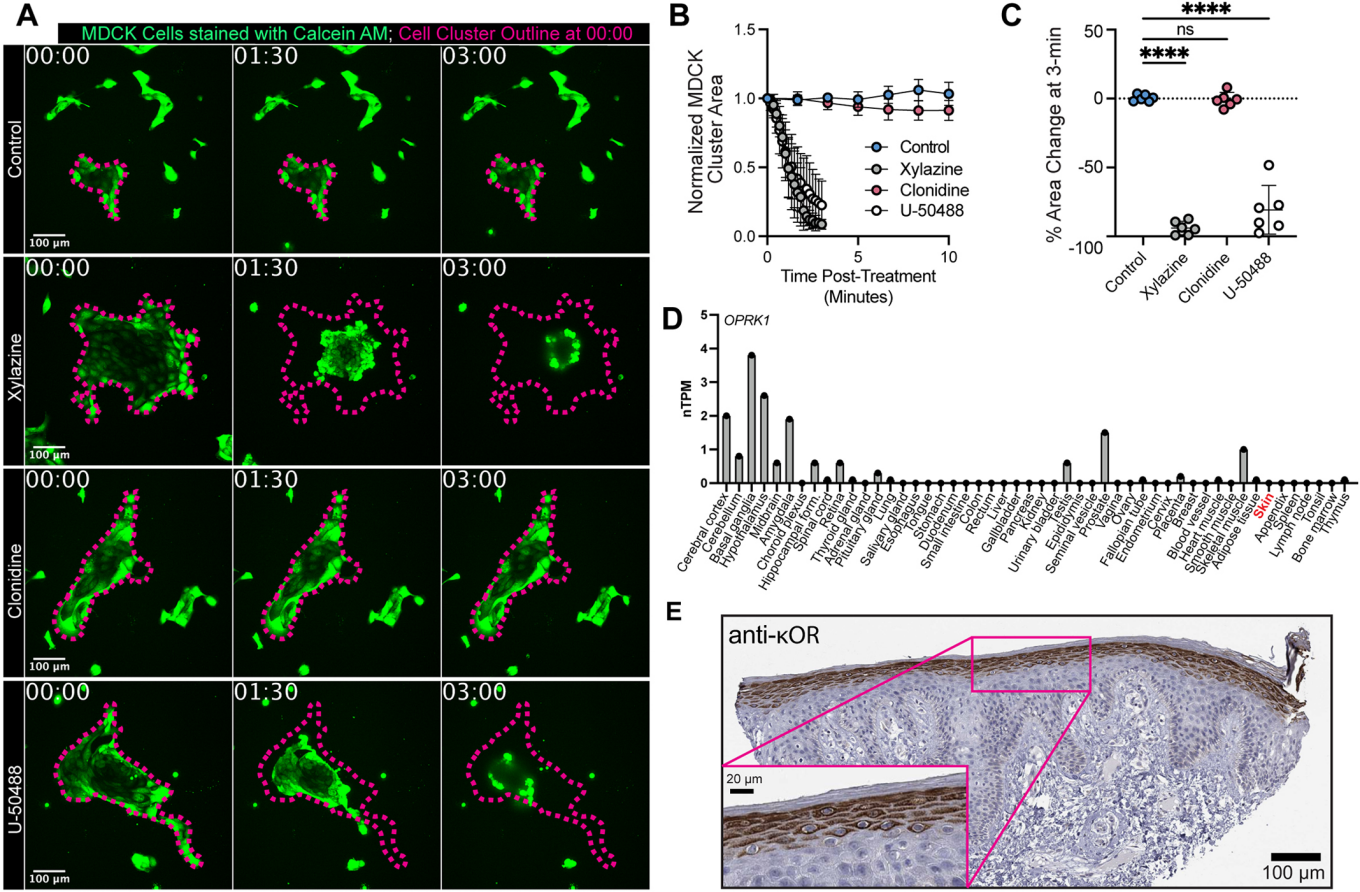
**Xylazine and U-50488 directly alter epithelial homeostasis.** (A) Timelapse imaging of MDCK cell clusters stained with Calcein-AM and subsequently treated with xylazine (5 mg/ml), clonidine (5 mg/ml), U-50488 (2 mM) or an equal volume of reverse-osmosis water (control), showing reduction of cluster 2D area. The cluster outline at the time of treatment is shown in magenta in all frames to highlight the changes in area. Time in mm:ss. Scale bars: 100 µm. (B,C) Quantification of normalized 2D MDCK cell cluster area from three independent experiments (*n*=6 total MDCK clusters; two clusters from independent wells per experiment) following the treatments described in A either over time (B) or at 3 min post-treatment (C). The average change in 2D area in each condition at 3 min post-treatment was compared to control using an ordinary one-way ANOVA with Dunnett’s correction for multiple comparisons. ns, not significant; ****P(control versus xylazine and control versus U-50488)<0.0001. Data are presented as mean±s.d. (D) Human *OPRK1* (κOR) mRNA expression across tissues from the Human Protein Atlas (proteinatlas.org). Expression shown as normalized transcripts per million (nTPM). Skin is highlighted in red. (E) Immunohistochemistry of κOR protein in human skin from the Human Protein Atlas (antibody HPA067549), showing **κ**OR expression in epidermal keratinocytes. Scale bars: 100 µm and 20 µm (inset).

To test this hypothesis, we expanded the Madin-Darby canine kidney (MDCK) epithelial cell line (Leighton et al., 1969) until they formed multicellular clusters, treated them with xylazine or an equal volume of RO water (control) and monitored the clusters over time. Mirroring the result observed in larval zebrafish tails (Fig. 2D-F, Fig. 3C,D), we found that xylazine resulted in a rapid contractile response in these epithelial cell clusters characterized by a significant drop in the 2D area (Fig. 4A-C; Movie 5). Just as we observed with larval zebrafish (Fig. 3C,D), we found that the κOR agonist U-50488 drove a similar contractile response (Fig. 4A-C; Movie 5). In contrast, the α2AR agonist clonidine had no significant effect on these epithelial cell clusters (Fig. 4A-C; Movie 5). We attempted to block xylazine-induced contraction by pretreating the MDCK cells with the κOR antagonist nor-binaltorphimine (nBNI) (Portoghese et al., 1987) but found that it had no effect on MDCK cell cluster contraction following xylazine treatment (Fig. S3A,B). While initially surprising, this result likely reflects the fact that xylazine is structurally different from classical κOR agonists such as U-50488 and may therefore engage the receptor through distinct mechanisms that are not sensitive to antagonists like nBNI. Indeed, although the evidence that xylazine binds to κOR is strong (Bedard et al., 2024; Floresta et al., 2025), the exact nature of this binding and how it compares to traditional agonists remains poorly understood.

Our data collectively point to xylazine acting directly on keratinocytes through κOR, and we therefore investigated whether κOR is expressed by human epidermal keratinocytes by examining the Human Protein Atlas (Karlsson et al., 2021; Uhlén et al., 2015; Uhlen et al., 2017). Whereas *OPRK1* mRNA expression was shown to be minimal in skin tissue by RNA sequencing (Fig. 4D), immunohistochemistry of human samples revealed robust κOR protein expression specifically in epidermal keratinocytes (Fig. 4E). Notably, the Human Protein Atlas antibody staining summary highlights skin as one of only two tissue types among >40 analyzed showing notable κOR expression: “Moderate to strong cytoplasmic positivity was observed in neuronal cells and skin, additional weak immunoreactivity was observed in neuronal cells in the CNS. Remaining tissues were mainly negative” (Karlsson et al., 2021; Uhlén et al., 2015; Uhlen et al., 2017). This high constitutive κOR protein expression in keratinocytes may, therefore, explain the vulnerability of the skin following exposure to a κOR agonist like xylazine (Bedard et al., 2024; Floresta et al., 2025).

## DISCUSSION

Together, these data point to a possible alternative mechanism by which xylazine causes skin damage in humans. Our findings suggest that xylazine, acting directly on epithelial cells through κOR, disrupts keratinocyte organization and intercellular junctions, resulting in tissue damage. Consistent with our hypothesis that activation of κOR is major driver of skin wounds following xylazine exposure, non-medical use of a separate κOR agonist called pentazocine is associated with skin lesions similar to the ones associated with xylazine use (Kumar et al., 2018; Palestine et al., 1980; Parks et al., 1971; Sahu et al., 2020; Schlicher et al., 1971). Further, injection of desomorphine (‘Krokodil’), a mu opioid receptor agonist that also acts as a weak kappa opioid receptor agonist, is associated with severe skin necrosis similar to that seen in patients with a history of xylazine injection (Katselou et al., 2014; Shelton et al., 2015). Together, these data suggest that κOR is a critical player in skin homeostasis and the wound healing response in humans.

We acknowledge that the xylazine concentration used in this study (5 mg/ml) is significantly higher than the blood concentrations measured in human case reports, which range from 0.0000033 to 0.002755 mg/ml in fatal overdoses (Hays et al., 2024). However, xylazine-induced wounds typically occur at injection sites, especially when veins are inadvertently missed (Malayala et al., 2022; McNinch et al., 2021), which exposes the surrounding tissue to the undiluted concentration of xylazine present in the injection solution itself, not the systemic blood levels that are commonly measured in the literature. Although we have not been able to find the concentration of xylazine found in the xylazine–fentanyl mixtures that have become prevalent over the past decade, there are numerous case studies in the literature in which users are either presumed or known to have directly injected veterinary-grade formulations of 100 mg/ml xylazine hydrochloride (Carruthers et al., 1979; Mittleman et al., 1998; Morris and Hoang, 2024; Spoerke et al., 1986). These studies suggest that the concentration of xylazine at the injection site of humans, at least in some instances, is 20-fold higher than the concentration used in this study and indicate that 5 mg/ml is well within the relevant range.

The rapid keratinocyte extrusion elicited by both xylazine and clonidine (Fig. 3A,B) was highly unexpected, as α2AR is not directly linked to epithelial cell extrusion to our knowledge. Epithelial cell extrusion is used to expel cells from the tissue without risking gaps in the barrier tissue, and it is a normal process both in development and homeostasis (Grata and Levayer, 2025; Rosenblatt et al., 2001). It can also be used to eliminate infected or transformed cells, and dysregulated cell extrusion may contribute to inflammatory disorders (Bastounis et al., 2021; Blander, 2016; Lawrence et al., 2022; Levayer, 2024; Moshiri et al., 2023; Tanimura and Fujita, 2020). Understanding the signaling cascades that prompt cell extrusion is, therefore, of clinical importance. We did not observe extrusion downstream of either xylazine or clonidine in our MDCK cell experiments *in vitro* (Fig. 4A-C; Movie 5), which may indicate that α2AR activation is acting indirectly to drive extrusion. Here, we focused on the tissue-wide contraction and basal keratinocyte disruption that occurs downstream of xylazine treatment, as these seem to have a more profound impact on skin homeostasis in our experiments. However, the process by which α2AR activation leads to rapid epithelial cell extrusion should be the focus of future studies.

Our data are not mutually exclusive with the prevailing hypothesis that xylazine-induced skin damage is the result of peripheral vasoconstriction through α2AR activation. It is possible that α2AR-mediated vasoconstriction and κOR-mediated tissue contraction are additive or synergistic responses in the development of skin lesions in human patients following xylazine exposure. Although drugs targeting κOR such as xylazine can disrupt skin homeostasis, the endogenous role of κOR in skin homeostasis and a healthy wound healing response remains poorly understood. κOR and its endogenous ligand dynorphin A are homeostatically expressed by keratinocytes, and κOR knockout mice show altered epidermal responses in a dry skin dermatitis model (Bigliardi-Qi et al., 2007; Dalefield et al., 2022; Tominaga et al., 2007). Although dysregulated κOR activation in the case of xylazine or pentazocine activation disrupts skin homeostasis, these data raise the possibility that the κOR pathway could be therapeutically targeted in the context of skin disorders and chronic wounds in the future.

## MATERIALS AND METHODS

### Zebrafish husbandry and maintenance

All protocols using zebrafish in this study were approved by the University of Wisconsin-Madison Research Animals Resource Center (protocol M005405-A02). Adult AB strain fish and previously published transgenic reporter lines Tg(*krt4*:UtrCH-GFP)^uwm53Tg^ (Lam et al., 2015), Tg(*krt4*:LifeAct-mRuby)^uwm54Tg^ (Lam et al., 2015), Tg(*krtt1c19e*:acGFP) (Lee et al., 2014), Tg(*krtt1c19e*:LifeAct-mRuby)^uwm55Tg^ (Fister et al., 2024) were used in this study. After breeding, fertilized embryos were placed into E3 medium (5 mM NaCl, 0.17 mM KCl, 0.44 mM CaCl_2_, 0.33 mM MgSO_4_, 0.025 mM NaOH and 0.0003% Methylene Blue) and maintained at 28.5°C in a Petri dish in a laboratory incubator until 5 dpf, when they were used for experiments.

### Agonist, antagonist and inhibitor preparation

Xylazine hydrochloride (Chem Cruz, sc-220393), clonidine hydrochloride (Sigma-Aldrich, C7897), (±)-U-50488 hydrochloride (Tocris, 0495) and nor-binaltorphimine dihydrochloride (nBNI; Sigma-Aldrich, N1771) were purchased in powder form, reconstituted in RO water at 50 mg/ml for xylazine hydrochloride, 50 mg/ml for clonidine hydrochloride, 20 mM for U-50488 hydrochloride and 20 mM for nBNI, and stored at −20°C until use. *Para*-aminoblebbistatin (Cayman, 22699) was dissolved in DMSO at a concentration of 200 mM and stored at −20°C until use.

### Zebrafish handling

For survival and initial skin damage experiments (Fig. 1), larvae at 5 dpf in E3 medium were anesthetized by the addition of tricaine (MS222/ethyl 3-aminobenzoate; Sigma-Aldrich) dropwise to a final concentration of ∼0.2 mg/ml. Once immobile, larvae were carefully added to 60 mm×15 mm dishes (Corning, 430166) containing the indicated concentration of xylazine dissolved in E3 medium. After 5 min or at the indicated time for Fig. 1B, larvae were gently transferred to a Petri dish with clean E3 medium. For calculating survival and skin damage, fish were maintained in clean E3 medium for 18 h, and survival was determined based on whether larvae had a detectable heartbeat by microscopy at this time. Larvae were then re-anesthetized in tricaine at 0.2 mg/ml, and, once they were non-motile, warm 2% low-melting-point agarose (Thermo Fisher Scientific, A-204-25) was mixed into 0.2 mg/ml tricaine E3 medium, and anesthetized zebrafish were gently added to the bottom of a 50-mm glass-bottom chamber (MatTek). Zebrafish were visually monitored as the agarose gelled, and an eyelash brush was used to keep the fish flat during this process. These fish were subsequently imaged to quantify skin damage. For the 24-well plate swim assay to measure sedation, we added tricaine dropwise just to the point at which larvae become non-motile and transferred fish to E3 medium containing xylazine or an equal volume of RO water. After 5 min, fish were then immediately transferred to the wells of a 24-well plate (Corning, 3537) containing fresh E3 medium and imaged within 1 h of the initial xylazine exposure. For timelapse imaging in response to xylazine, clonidine or U-50488, larvae were mounted in a zWEDGI restraining device (Huemer et al., 2017) with the head immobilized in 2% low-melting-point agarose, and the tail was left unrestrained and exposed to the surrounding medium. For brightfield imaging in response to agonist treatment, fish were imaged for 5-10 min in E3 medium with 0.2 mg/ml tricaine, and the agonist was gently added into the side of the chamber at the ‘00:00’ timepoint to the indicated final concentration. As a control, we added an equal volume of RO water, the solvent used for dissolving all agonists used in this study, to the chamber. For fluorescence microscopy, we added the agonists, refocused the objective and began imaging within ∼10 s of treatment.

### Microscopy and image preparation

All brightfield imaging was performed on a Zeiss Zoomscope (EMS3/SyCoP3; 1× Plan-NeoFluar Z objective) with an Axiocam Mrm charge-coupled device camera using ZenPro 2012 software (Zeiss). Most fluorescence microscopy was performed using a spinning disk confocal microscope (CSU-X, Yokogawa) with a confocal scanhead on a Zeiss Observer Z.1 inverted microscope equipped with either an EC Plan-Neofluar 10×/0.30 objective, a Plan-Apochromat 20×/0.8 NA objective, an EX Plan-Neofluar 40×/0.75 objective or a Plan-Apochromat 63×/1.4 NA objective, Photometrics Evolve EMCCD camera and Zen software (Zeiss). The remaining fluorescence microscopy was performed on a Nikon Eclipse Ti2 microscope equipped with a Crest X-light V3 spinning disc unit and Kinetix22 monochrome camera. All imaging was performed using a Plan Apochromat 60×/1.42 NA objective, with a Tokai Hit stage top incubation system used for imaging cultured MDCK cells. Images were collected using NIS-Elements AR 6.10.01 software. For tracking larvae swim distances, movies were imported into ImageJ (v. 2.1.0/1.53c), and larvae were tracked using the Manual Tracking plugin. For quantifying the area of the post-anal fin fold, post-anal notochord and somite area, or MDCK cell clusters, we manually outlined the region of interest using the ImageJ polygon tool and measured the area. For counting keratinocyte extrusion events, we analyzed all movies frame by frame (1 min interval) and enumerated the cumulative number of events over the course of 30 min post-treatment. We excluded the tip of the caudal fin from analysis as this region often curled upwards; the analyzed region is shown above the relevant graphs.

### MDCK cell culture

MDCK cells were originally obtained from American Type Culture Collection and had been maintained in liquid nitrogen since 2003. These cells were not recently authenticated or tested for contamination but displayed the characteristic epithelial morphology and behavior typical of this cell line. MDCK cells were cultured in Dulbecco's modified Eagle medium (DMEM)/F12 medium supplemented with 5% fetal bovine serum (FBS) and 1% penicillin–streptomycin antibiotic solution. MDCK cells were cultured on a 10-cm tissue culture plate until ∼80% confluency and then lifted using TrypLE EDTA solution and reseeded onto Ibidi eight-well glass-bottom chambers for imaging. After adhesion of MDCKs on the Ibidi chambers, cells were stained with Calcein-AM intracellular dye (1:1000) for 20 min. The Calcein-AM was washed off, and cells were put back into DMEM/F12 medium until treatment with xylazine hydrochloride, U-50488 or clonidine hydrochloride and subsequent imaging. For experiments with nBNI, the cells were treated with the antagonist at 10 µM or an equal volume of RO water for 1 h prior to treatment with xylazine, and the xylazine solution that was added to the cells also had 10 µM nBNI (or an equal volume of water) such that the concentration of the antagonist stayed consistent throughout the experiment.

### Statistical analysis

Statistical tests were all performed using GraphPad Prism (version 9.4.1), with specific tests indicated in each figure legend. For experiments with three or more larvae per condition per experiment, nested *t*-tests were used to account for within-experiment correlation. Comparisons of multiple groups to a single control were performed using ordinary one-way ANOVA with Dunnett's correction for multiple comparisons. For experiments with small sample sizes (*n*=3), the *t*-test was chosen over non-parametric alternatives because the Mann–Whitney *U* test cannot achieve *P*<0.1 at this sample size regardless of effect magnitude, whereas the *t*-test is robust to violations of normality with equal sample sizes and large effect sizes. *P*-values are denoted in each graph, with ‘ns’ indicating *P*>0.05.

## Supplementary Material

10.1242/dmm.052600_sup1 Supplementary information
