# Supplementary material for: Evidence that xylazine disrupts skin homeostasis by acting on epithelial cells through the kappa opioid receptor
Source: Dis Model Mech. 2026 Mar 31;19(3):dmm052600. doi: 10.1242/dmm.052600 (PMC13072079; doi:10.1242/dmm.052600)
Supplement: Supplementary information [file dmm-19-052600-s1.pdf]

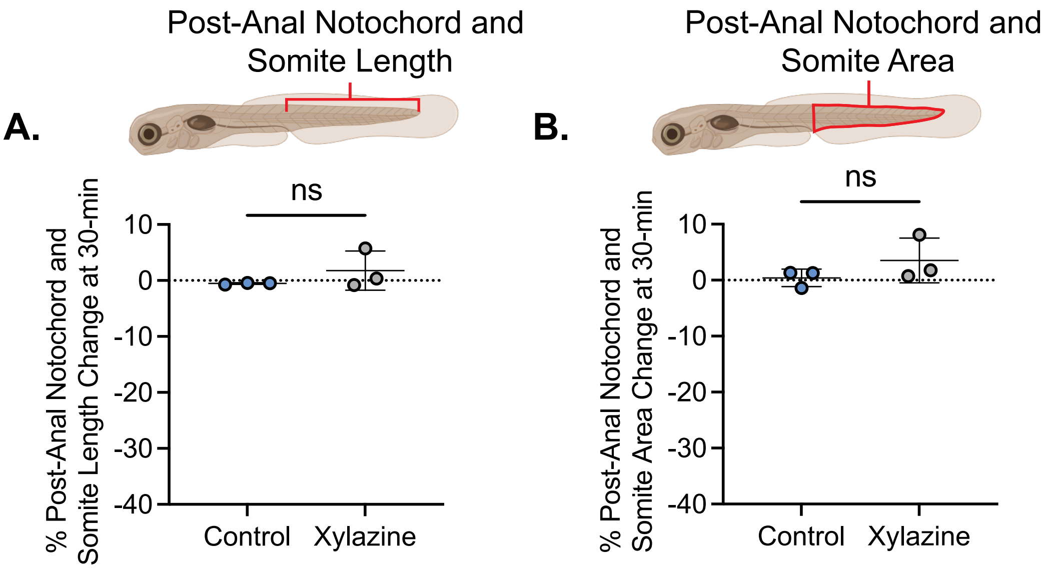

Fig. S1. Xylazine treatment does not affect zebrafish notochord length or area. A-B. The change in the length (A) and 2D area (B) of the notochord and somite region posterior to the anus from 3 independent experiments (n = 3 total larvae, 1 larva per experiment). Groups were compared using two-tailed Welch's t-tests; neither comparison was statistically significant (ns). The diagrams denote the region that was analyzed. Data are presented as mean±s.d.

Disease Models & Mechanisms • Supplementary information

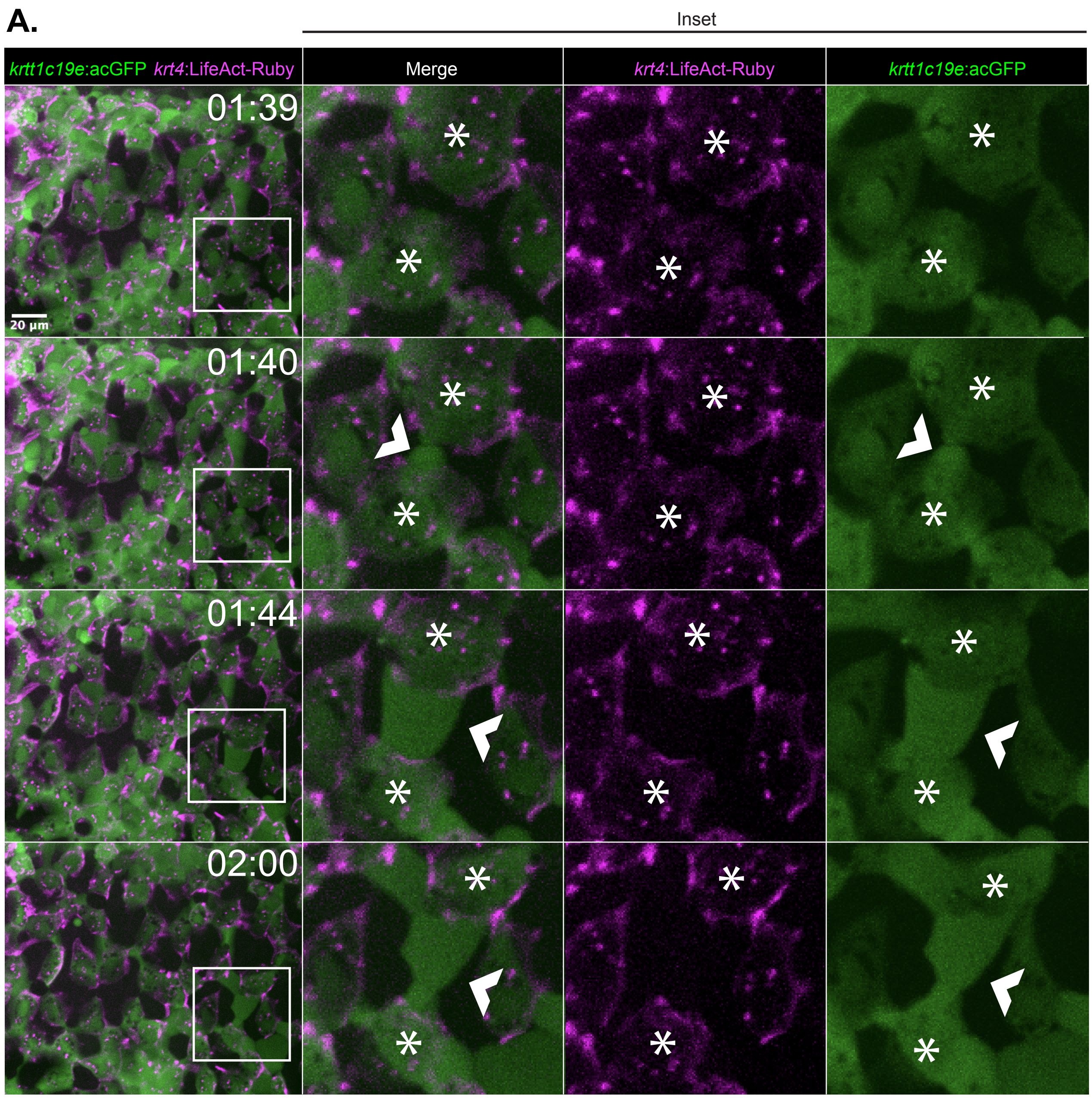

Fig. S2. De-adhered keratinocytes develop F-actin-poor cytoplasmic contacts with neighboring keratinocytes.

A. Timelapse imaging of basal keratinocyte GFP and keratinocyte LifeAct in tg(krtt1c19e:acGFP; krt4:LifeAct-mRuby) zebrafish larvae following treatment with xylazine (5 mg/mL). This movie follows the fate of basal keratinocytes after they have de-adhered from their neighbors (like in Fig. 2H). The time (hh:mm) post-xylazine treatment is noted in the left column images. In the inset images, the asterisks mark two de-adhered keratinocytes, and, between 1:40-2:00, cytoplasmic GFP (white arrowhead) that extends past any visible LifeAct can be seen forming a contact between these two cells. In the larger image, especially at 2:00, the broad formation of these types of contacts can be seen. Representative of three independent experiments (n = 3 total larvae, 1 larva per experiment). Time = hh:mm. Scale bar = 20 µm.

Disease Models & Mechanisms • Supplementary information

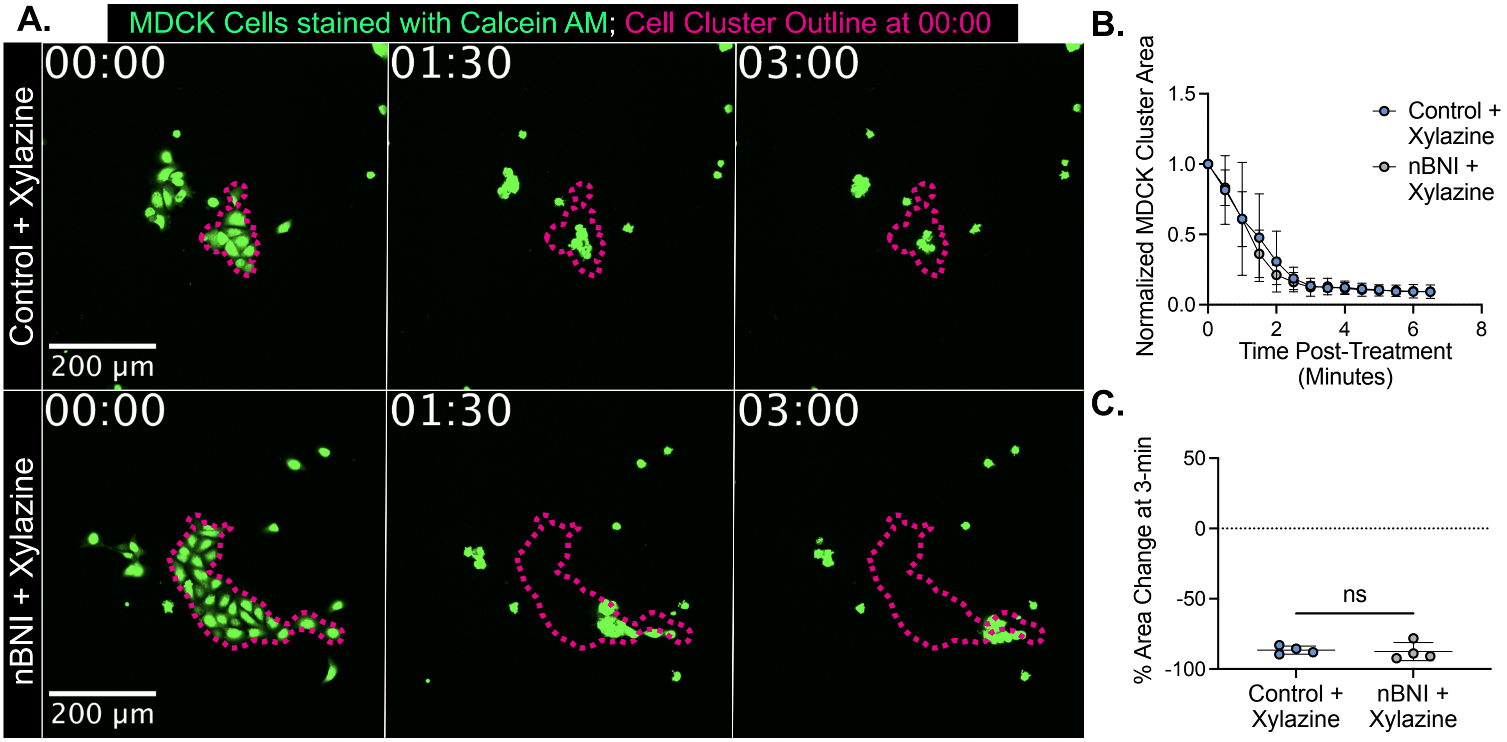

Fig. S3. The κOR antagonist nBNI does not alter MDCK cluster contraction following xylazine treatment.

A. Timelapse imaging of MDCK cell clusters stained with calcein AM pretreated with 10 uM nBNI for 1-hour or vehicle (control) and subsequently treated with xylazine (5 mg/mL) showing the drop in 2D area over time, with the magenta outline highlighting the cluster area at 0 minutes post-treatment. Time in mm:ss, scale bar = 200 μm. **B-C.** Quantification of normalized 2D MDCK cell cluster area from 2 independent experiments (n = 4 total MDCK clusters, 2 clusters per experiment) following the treatments described in (A) either over time (B) or at 3-minutes post-treatment (C). The average change in 2D area in each condition at 3-minutes post-treatment was compared to control using an ordinary one-way ANOVA with the Dunnett correction for multiple comparisons (ns). Data are presented as mean±s.d..

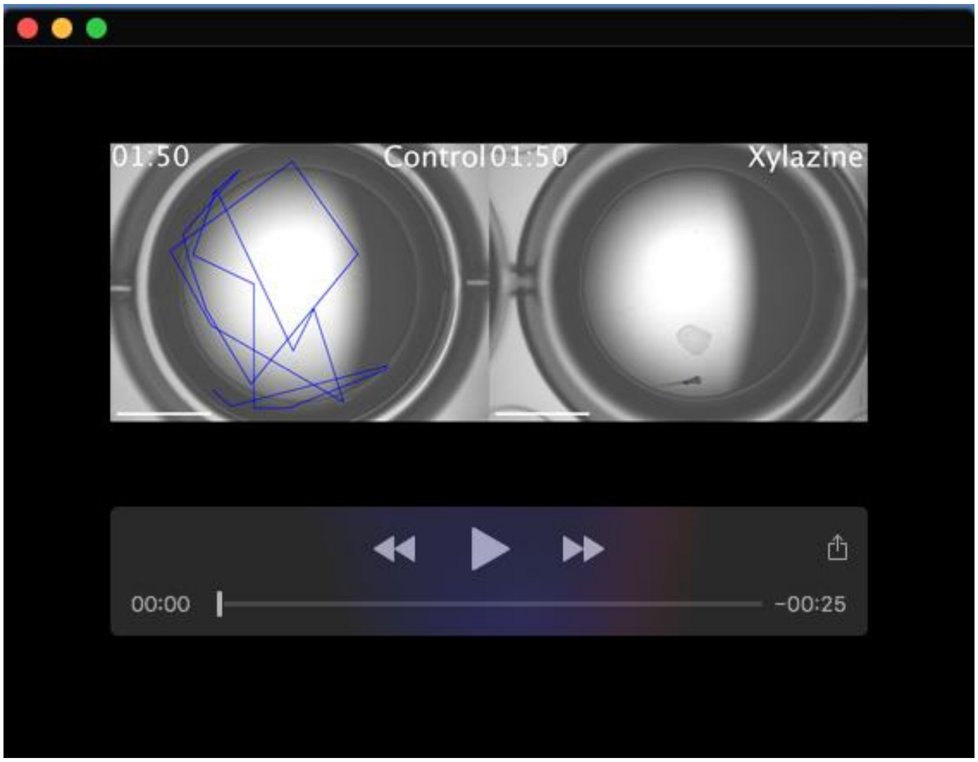

Movie 1. Xylazine treatment causes sedation in larval zebrafish.

Following treatment with xylazine or an equal volume of water added to E3 (control), larvae were moved to 24-well plate with fresh E3 and imaged over the course of 5 minutes to quantify their movement, which is highlighted with the overlaid blue track. All imaging took place within 1-hour of xylazine treatment. Time in mm:ss, Scale bar = 0.5 cm.

Disease Models & Mechanisms • Supplementary information

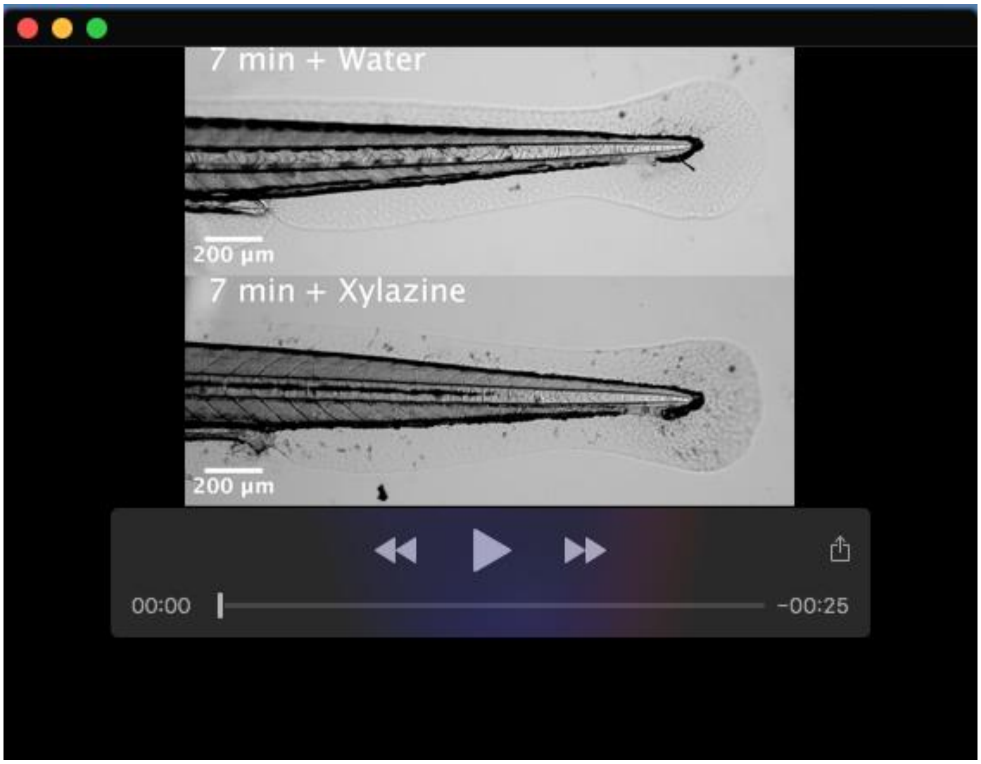

Movie 2. Xylazine treatment causes keratinocyte extrusion and tissue-wide contraction.

Zebrafish larvae were mounted in a zWEDGI restraining device in E3 with 0.2 mg/mL tricaine and imaged by brightfield microscopy for 5 minutes, at which time xylazine (bottom panel) or an equal volume of RO water (top panel) was dripped into the chamber to a final volume of 5 mg/mL xylazine. Note the keratinocyte extrusion events that occur between 3-10 minutes post-treatment with xylazine and the tissue-wide tail contraction that occurs throughout the movie. Scale bars = 200 μm.

Disease Models & Mechanisms • Supplementary information

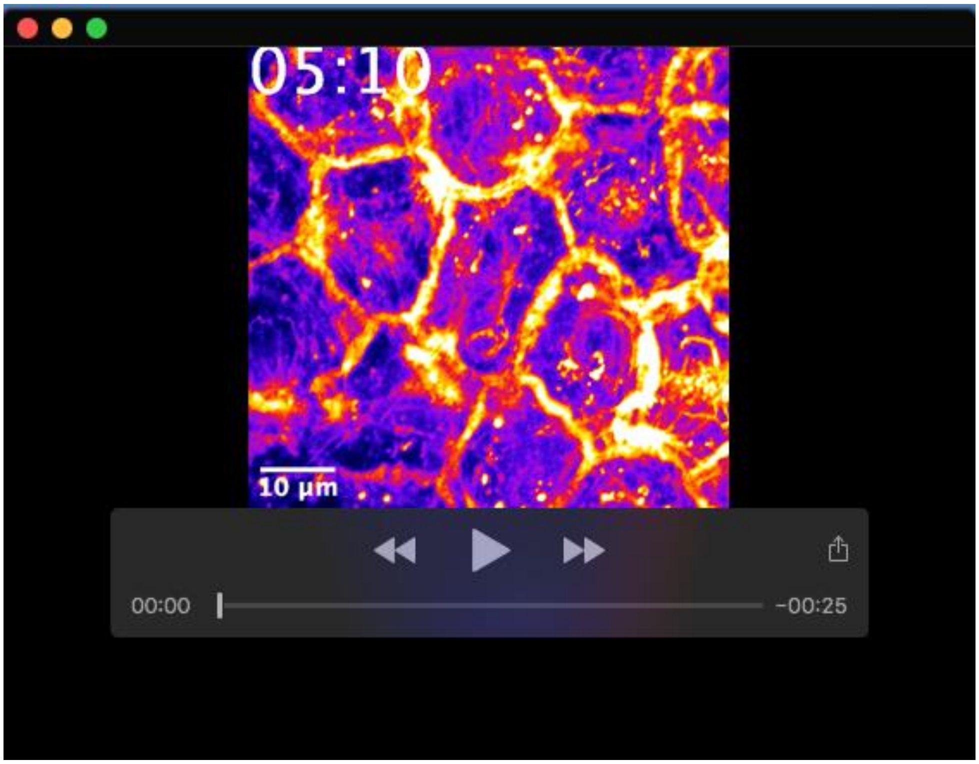

Movie 3. Xylazine treatment leads to the disruption of basal keratinocyte interactions.

A zebrafish transgenic reporter line larvae in which basal keratinocytes express LifeAct (tg(*krtt1c19e*:LifeAct-mRuby)) was mounted in a zWEDGI restraining device in E3 with 0.2 mg/mL tricaine, treated with xylazine to a final concentration of 5 mg/mL, and immediately imaged by spinning disc confocal microscopy. Clear intercellular adhesions apparent at the beginning of the movie between basal keratinocytes become progressively disorganized. Note this movie was refocused at the 9-minute mark. Time in mm:ss, scale bar = 10 μm.

Disease Models & Mechanisms • Supplementary information

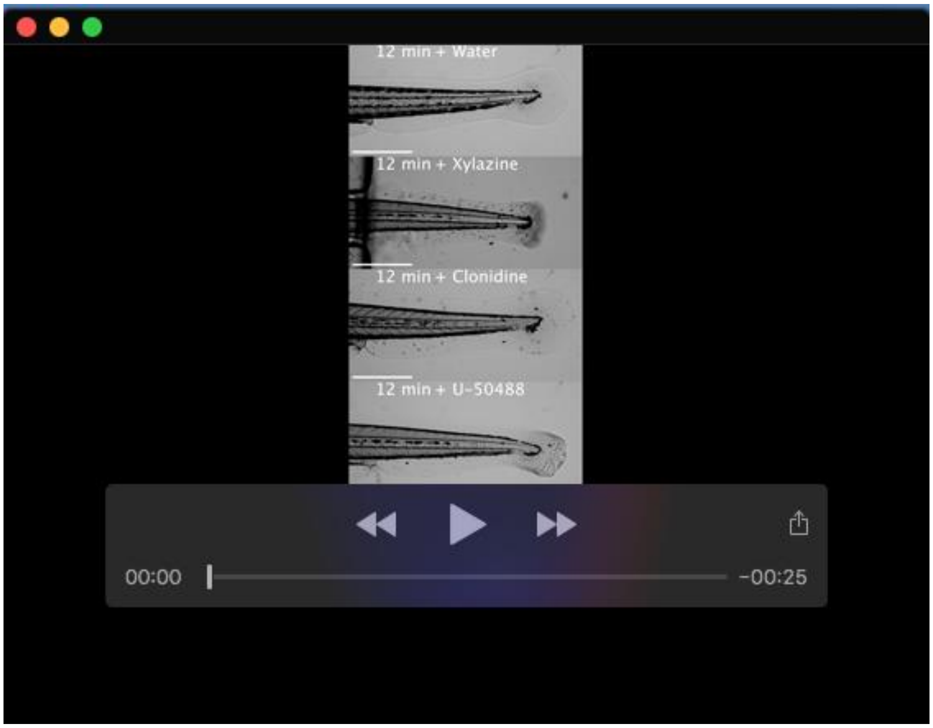

Movie 4. Xylazine-induced extrusion and tissue-wide contraction can be replicated with α2AR and κOR agonists, respectively.

Zebrafish larvae at 5 dpf were immobilized in a zWEDGI restraining device in E3 with 0.2 mg/mL and imaged following the addition of xylazine (5 mg/mL final), clonidine (5 mg/mL final), U-50488 (1 mM final), or an equal volume of water as a control. Note that keratinocyte extrusion and contraction are apparent in the xylazine-treated larvae, but only extrusion is apparent in clonidine-treated larvae. Further, U-50488 does not cause any significant extrusion but does cause considerable tissue contraction. Scale bars = 500 μm.

Disease Models & Mechanisms • Supplementary information

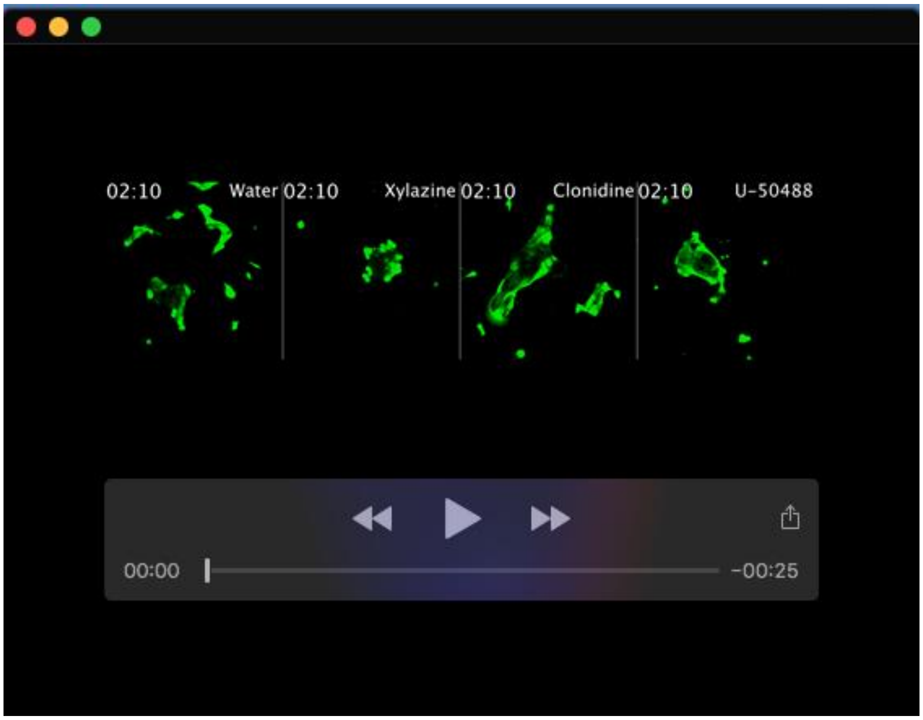

Movie 5. Xylazine and U-50488 act directly on epithelial cells to cause contraction. MDCK cells were expanded to the point that they formed small multicellular clusters, stained with calcein AM and treated with xylazine (5 mg/mL final), clonidine (5 mg/mL final), U-50488 (1 mM final), or an equal volume of water as a control. Note the rapid cell cluster contraction that occurs following treatment with xylazine and U-50488 but not clonidine or water. Time in mm:ss, scale bars = 100 μm.
